# Supplementary material for: Subgap spectroscopy along hybrid nanowires by nm-thick tunnel barriers
Source: Nat Commun. 2023 Oct 20;14:6647. doi: 10.1038/s41467-023-42422-z (PMC10589238; doi:10.1038/s41467-023-42422-z)
Supplement: Supplementary file 1 — Supplementary Information [file 41467_2023_42422_MOESM1_ESM.pdf]

Supplementary Information: Subgap spectroscopy along hybrid nanowires by nm-thick tunnel barriers

Vukan Levajac[1*], Ji-Yin Wang[1,2*†],
Cristina Sfiligoj[1], Mathilde Lemang[1], Jan Cornelis Wolff[1], Alberto Bordin[1],
Ghada Badawy[3], Sasa Gazibegovic[3], Erik P. A. M. Bakkers[3],
Leo P. Kouwenhoven[1]

[1]QuTech and Kavli Institute of Nanoscience, Delft University of Technology,
2628GA Delft, The Netherlands
[2]Beijing Academy of Quantum Information Sciences,
100193 Beijing, China
[3]Department of Applied Physics, Eindhoven University of Technology,
5600MB Eindhoven, The Netherlands
[*]These authors contributed equally to this work.
[†]To whom correspondence should be addressed; E-mail:
wangjiyinshu@gmail.com.

Device fabrication

In this work, intrinsic Si wafers covered with $285\,\mathrm{nm}$ $\mathrm{SiO_2}$ were used as substrates. On top of the $\mathrm{SiO_2}$ layer, gates were lithographically defined and grown by depositing $3/17\,\mathrm{nm}$ Ti/Pd in an electron-beam evaporator. After that, atomic layer deposition (ALD) was used to grow $\sim 20\,\mathrm{nm}$ high-quality $\mathrm{HfO_2}$ at $110°$C to serve as the gate dielectric. Next, shadow-walls were defined on top of the $\mathrm{HfO_2}$ layer. In this step, FOx-25 (HSQ) was first spun at $1.5\,\mathrm{krpm}$ for 1 min and hot-baked at $180°$C for 2 min. Then, the HSQ layer was lithographically patterned and developed with MF-321 at $60°$C for 5 min. After the formation of the HSQ shadow-walls, stemless InSb

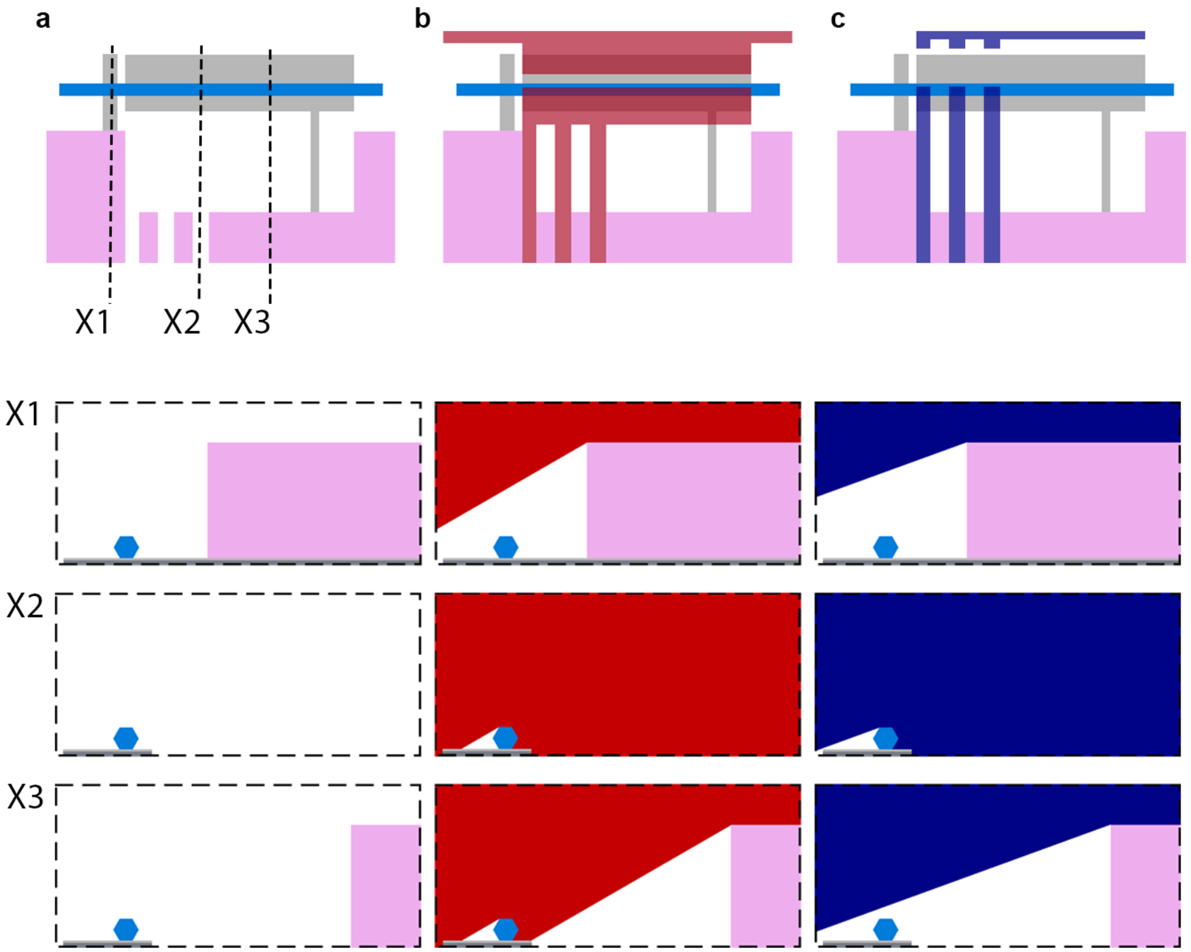

Figure S1: **Device fabrication by shadow-wall lithography. a** A schematic representation of the top view on a substrate with a nanowire (light blue), gates (grey) and shadow-walls (lilac). Dashed black lines denote three transverse cross sections X1, X2 and X3 depicted below. **b** A schematic representation of the top view on the substrate and X1, X2 and X3 cuts for the Al (red) deposition at 30° with respect to the substrate. **c** Analogous to (b), but for the Ag (navy) deposition at 18° with respect to the substrate. Due to the smaller angle, the shadow-walls create longer shadows during the Ag deposition and three probes are selectively defined along the nanowire.

nanowires were precisely deposited on top of the gates by an optical nano-manipulator. Fig. S1a displays the nanowire (light blue), gates (grey) and shadow-walls (lilac) before further

fabrications.

Figure S1b shows the deposition of the superconducting Al film. In this step, several substeps were carried out. First, the native oxide on the surface of the InSb nanowire was removed by a gentle hydrogen cleaning. Then, Al film was grown at a temperature of $140\,\mathrm{K}$. The Al was deposited at an angle of 30° with respect to the substrate and with a flux of $5.5\,\mathrm{nm}$ (this is an aimed value and the actual thickness can be different, see Fig. S2). Due to the hexagonal nanowire cross-section and the specific deposition angle, three facets of the nanowire are covered with Al. As the direction of the Al flux is perpendicular to one facet, the thickness of the Al film on this facet corresponds to the flux. The Al flux forms angles of 30° with other two facets and the substrate. Consequently, the Al film thickness there is half of the flux. The Al growth was followed by in-situ oxidation in the load lock chamber of the evaporator. Here, the Al film was oxidized for 10 min at $10\,\mathrm{Torr}$ oxygen pressure. This was precisely controlled such that the Al film on one nanowire facet (where it is thicker) is partially oxidized and the Al film on the other two nanowire facets (where it is thinner) and on the substrate is fully oxidized. Therefore, the superconducting Al remains only on one nanowire facet. On this facet, it is covered by the thin dielectric AlOx layer which continuously extends over the other two nanowire facets and the substrate - as the Al there has been completely turned into AlOx (see Fig. S2). The full oxidation of the Al on the substrate was additionally confirmed by measuring high resistance ($\sim\ \mathrm{G}\Omega$) of Al films on chips without nanowires after the same Al deposition and in-situ oxidation steps. The thin AlOx layer on top of the InSb-Al nanowire would serve as tunnel barriers for tunnel probes, which are fabricated in the next step.

After the oxidation in the load lock chamber, the sample was warmed up to room temperature and then it was inserted back into the evaporation chamber. As shown in Fig. S1c, $80\,\mathrm{nm}$ Ag was deposited at an angle of 18° with respect to the substrate. Due to the smaller deposition angle in comparison to the Al deposition at 30°, the shadow-walls cast longer shadows and

block the growth of Ag on the nanowire sections aligned with the shadow-walls. Consequently, Ag reaches the nanowire only through the interruptions in the shadow-walls, which determine the positions of the three Ag leads along the hybrid. These leads are grown on top of the previously formed AlOx layer and are used as probes P1, P2 and P3. The described Ag deposition was performed for Device 1 and 2. For Device 3, the probes P1, P2 and P3 are made of thick Al rather than Ag, while keeping all the other parameters unchanged.

A transmission electron microscopy (TEM) analysis of a transverse cross-section of a hybrid nanowire device with Ag leads is made and shown in Fig. S2. From the figure, we see that the device is composed of materials as designed. On the top and bottom-right facets of the nanowire, O appears where Al exists, indicating a fully oxidized Al layer on these two facets. For top-right facet, O only exists on the surface of the Al layer, leading to an oxidized layer on the surface. From the bottom three panels in Fig. S2, we observe that the atomic fraction of Al is always higher than that of O. Though the reason is not known yet, we are confident that the Al layer with O is oxidized enough to be insulating, as the planar SIN junction in Fig. 1f is fabricated with similar oxidation condition and the planar junction exhibits appealing tunneling spectroscopy results. Besides, we see that the element Ag appears in the Al region on the top-right facet and the reason is not known yet to us. In addition, we see that the thickness of the AlOx and Al layers in TEM cuts is a bit different from the designed values. However, the device functionality is not influenced.

The normal probe P0 and the drain contact were fabricated ex-situ after the growth of probes P1, P2 and P3. First, the two contacts were lithographically defined at the nanowire ends. Then, Ar ion milling was used to remove the native oxide (for the contact of probe P0) and the AlOx layer (for the drain contact), where also the Al can be affected by the Ar ion milling. Finally, $10/120\,\mathrm{nm}$ of Ti/Au was deposited in an electron-beam evaporation step followed by lift-off.

For the fabrication of the planar tunnel junction (Fig. 1f), a substrate with specifically

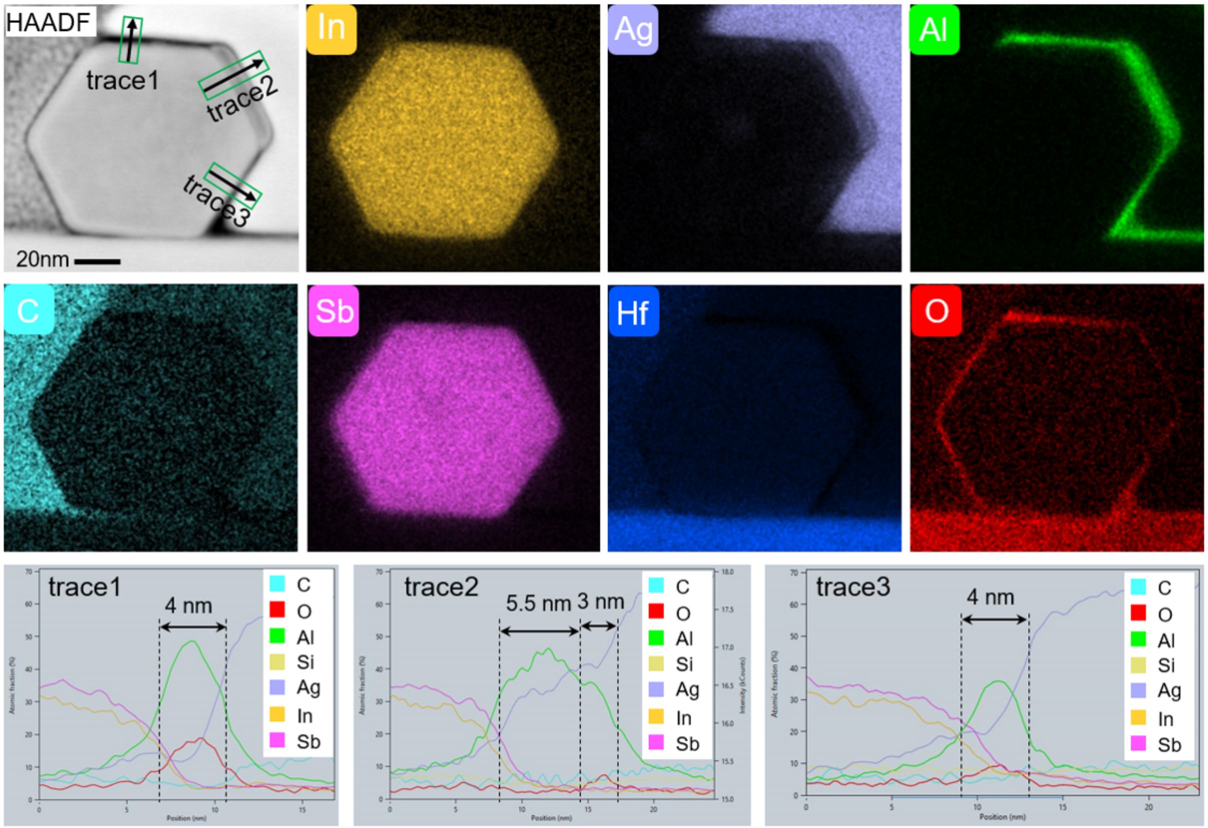

Figure S2: **Transmission electron microscopy (TEM) analysis of a transverse cross-section of a hybrid nanowire**. (1) The most left panel in the first row is the high-angle annular dark-field scanning transmission electron microscopy (HAADF STEM) image of the cross-section. Three linecuts of the integrated atomic fractions within the green boxes are shown in the third row. (2) The right three panels in the first row and the four panels in the second row display the energy-dispersive X-ray spectroscopy (EDX) composite maps of different elements, including In, Ag, Al, C, Sb, Hf and O. (3) In the third row, atomic fractions of different elements along the three traces are shown. Traces 1, 2 and 3 come from the top, top-right and bottom-right facet of the hybrid nanowire, respectively. The top and bottom-right facet both have $\sim 4\,\mathrm{nm}$ of Al, as well as $\sim 4\,\mathrm{nm}$ of O - indicating fully oxidized facets. For the top-right facet, the AlOx layer is a bit thinner ($\sim 3\,\mathrm{nm}$). The thickness of the Al layer without oxide is $\sim 5.5\,\mathrm{nm}$.

designed shadow-walls and without gates and nanowires was used. The Al film was deposited and then in-situ oxidized at the pressure of $1\,\mathrm{Torr}$. Next, the Ag film was deposited. See details in Ref. *(32)* cited in the main text.

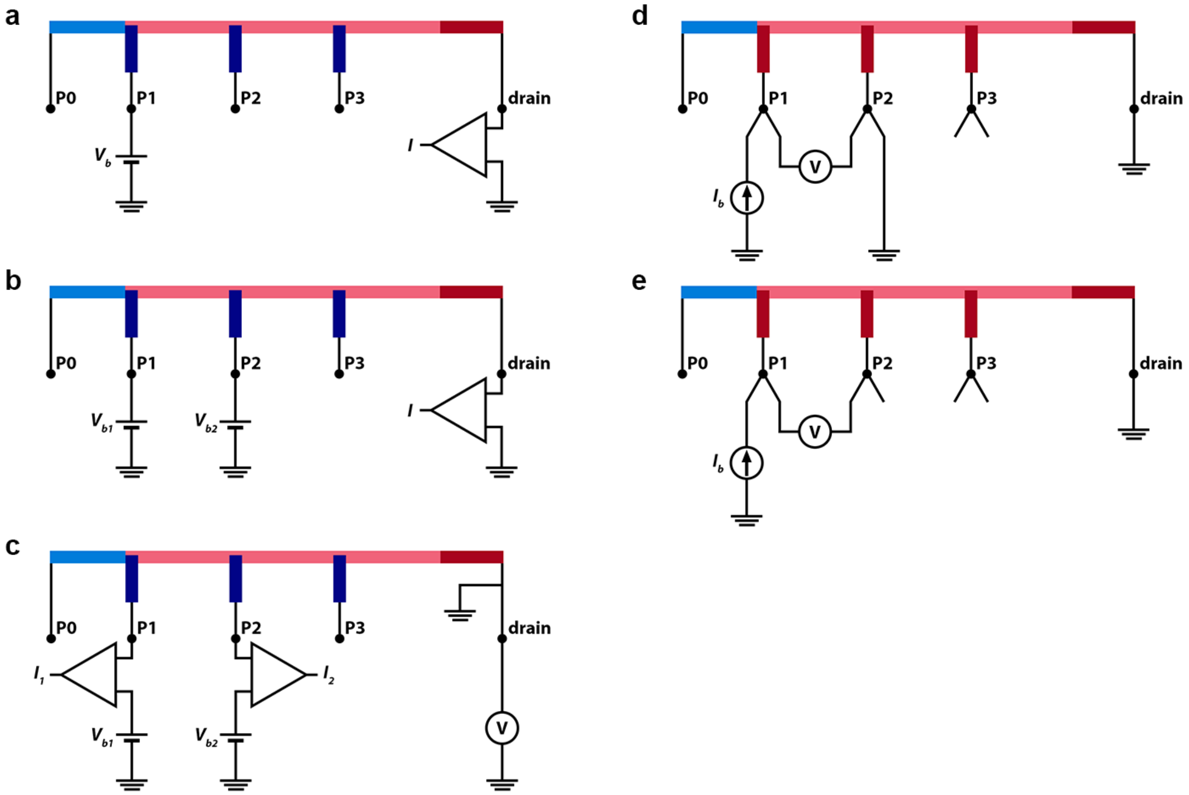

Figure S3: **Measurement setups**: Each panel contains a schematic representation of the nanowire device with the four probes P0, P1, P2 and P3 and the drain lead. Five panels represent the five measurement setups: (**a**) Setup V1, (**b**) Setup V2, (**c**) Setup V3, (**d**) Setup I1 and (**e**) Setup I2. For each setup, any probe or pair of probes can be chosen to be analogously connected as in the shown examples.

Measurement setups

The measurements were performed at a base temperature of $\sim 20\,\mathrm{mK}$ inside a dilution refrigerator equipped with a superconducting vector magnet. As shown in Fig. S3, three voltage-bias setups (V1, V2 and V3) are used for conductance measurements and two current-bias setups (I1 and I2) are used for supercurrent measurements. Table S1 summarizes the used measurement setups for each figure and corresponding serial resistance in each setup is provided as well. In the following paragraphs, we will discuss each measurement setup in Fig. S3 in detail.

Fig. \ Setup (R_s)	V1 ($8.89\,\mathrm{k\Omega}$)	V2 ($8.89\,\mathrm{k\Omega}$)	V3 ($5.81\,\mathrm{k\Omega}$)	I1	I2
1	■				
2	■				
3					■
4		■			
5A		■			
5B	■				
S4				■	
S5			■		
S6A	■				
S6B		■			
S7		■	■		
S8			■		
S9		■			
S10		■			
S11		■			
S12			■		
S13	■				
S14A	■				
S14B					■

Table S1: A summary of the used measurement setup for each figure and its serial resistance.

In the voltage-bias setups (V1, V2 and V3), dc-voltage sources are used to set dc-components of the bias voltages (V_b) and current-meters are used to measure dc-components of the currents (I). Lock-in amplifiers are used to apply ac-components of the bias voltages (dV_b with amplitudes of $10\,\mu$V) and measure ac-components of the currents (dI) - in order to obtain the differential conductance (G). The values of the dc- and ac-bias voltages are corrected for the voltage drops across a serial resistance R_s as $V_b \to V_b - IR_s$ and $dV_b \to dV_b - dIR_s$ (R_s for each setup is given in Table S1). The differential conductance at the dc bias voltage of $V_b - IR_s$ is $G = dI/(dV_b - dIR_s)$.

Setup V1 represents a two-terminal voltage-bias setup where a bias voltage $V_b + dV_b$ is applied to a single probe and a current $I + dI$ is measured in the drain contact. The three remaining probes are floating. Fig. S3a shows Setup V1 with the bias voltage applied to the probe P1. The serial resistance $R_s = 8.89\,\mathrm{k}\Omega$ includes the resistances of the two fridge lines and the series resistances of the voltage source, current-meter and low-pass filters on the PCB. When the differential conductance is high, even a slight overestimation of R_s may cause obtaining falsely negative G - due to the negative value of $dV_b - dIR_s$. This is the reason for the large negative values in the traces of Fig. 2. The setup can be applied analogously to any other probe.

In Setup V2 bias voltages $V_{b1} + dV_{b1}$ and $V_{b2} + dV_{b2}$ are applied to two probes and a current $I + dI$ is measured in the drain contact. The remaining two probes are floating. Fig. S3b shows Setup V2 with the bias voltages applied to the probes P1 and P2. Two lock-in amplifiers are used for applying dV_{b1} (lock-in1 at frequency f_1) and dV_{b2} (lock-in2 at frequency f_2). Upon setting a parameter value (magnetic field or gate voltage), the dc-bias voltages are consecutively swept on the two probes and the differential conductance is measured by the corresponding lock-in amplifier. For instance, V_{b1} is swept and the lock-in1 is used to measure the ac-current dI in the drain, while both V_{b2} and dV_{b2} are fixed at zero (P2 is an inactive probe in this case). Then, V_{b2} is swept and the lock-in2 is used to measure the ac-current dI in the drain, while both V_{b1}

and dV_{b1} are fixed at zero (P1 is an inactive probe in this case). Consequently, the inactive probe is effectively grounded and the ac-current dI in the drain has the frequency f_i while the dc-bias voltage V_{bi} is being swept ($i = 1, 2$). Grounding the inactive probe opens an additional channel for current that does not flow through the drain contact. Consequently, this could cause underestimations of the dc-current I and the ac-current dI. However, the additional channel has a resistance of the order of hundreds of kΩ (if probe P1, P2 or P3 is grounded), or even of the order of MΩ (if probe P0 is grounded), see the red traces in Fig. 2. These resistances are much higher than the resistance in the line of the drain contact (order of few kΩ). Therefore, the current in Setup V2 are predominantly drained by the drain contact and the underestimation due to the additional channel is negligible. This is additionally confirmed in Fig. S6, where the conductance measured by probe P1 does not change upon changing probe P0 from a pinch-off to a tunneling regime. Upon disconnecting P1 and P2 in Fig. S3b, the setup can be analogously applied to any other pair of probes.

In Setup V3 bias voltages $V_{b1} + dV_{b1}$ and $V_{b2} + dV_{b2}$ are applied to two probes and currents $I_1 + dI_1$ and $I_2 + dI_2$ are measured in these probes while the drain contact is connected to the cold-ground. The remaining two probes are floating. An additional voltmeter is used to detect the cold-ground fluctuation and correct the bias voltages. Fig. S3c shows Setup V3 with the bias voltages applied to the probes P1 and P2. Two lock-in amplifiers are used for applying dV_{b1} (lock-in1 at frequency f_1) and dV_{b2} (lock-in2 at frequency f_2). As in Setup V2, upon setting a parameter value (magnetic field or gate voltage), the dc-bias voltages are consecutively swept on the two probes and the differential conductance is measured by the corresponding lock-in amplifier. While the dc-bias voltage is swept at one probe, the other probe is kept effectively grounded, as explained for Setup V2. The serial resistance for each probe is $R_s = 5.81\,\mathrm{k}\Omega$, smaller than that in Setup V2, as the drain contact is connected to the cold-ground. Upon disconnecting P1 and P2 in Fig. S3c, the setup can be analogously applied to any other pair of

probes.

The use of Setup V2 and Setup V3 is motivated by an advantage to reliably examine the correlation behaviors between two probes. In contrast to Setup V1, the bias voltage is swept consecutively on both probes at each gate or magnetic field set point. This allows for a direct examination of the correlation between the subgap spectra in the two probes. Even if drifts in the device or setup are present, they appear at the same gate or field set point in both probes, and thus do not complicate the evaluation of the correlation in Setup V2 and Setup V3.

In spite of different setups being in use, we do not see that switching between different configurations influences the electrostatic environment. As it can be seen in Fig. S13, changing the setups does not affect the measured subgap spectra.

In the current-bias setups (I1 and I2), dc-current sources are used to set dc-bias currents (I_b) and voltmeters are used to measure dc-voltage drops (V). In order to allow for four-terminal configurations, each probe is connected to two fridge lines. Probe P0 is kept floating in the current-bias measurements.

In Setup I1 a bias current I_b is applied between two probes (or between one probe and the drain). A voltage drop V is measured between the two probes (or between the probe and the drain). The remaining probes are floating. Fig. S3d shows Setup I1 with the bias current applied between the probes P1 and P2, and the voltage drop measured across the series of two Josephson junctions (JJ1 of P1 and JJ2 of P2). Upon disconnecting P1 and P2 in Fig. S3d, bias current can analogously be applied in the same setup between any other pair of probes (or a probe and the drain). Note that if two probes are connected in the setup, a series of two JJs is measured, and if one probe and the drain are connected in the setup, a series of a single JJ and the drain contact is measured. If the drain contact has a finite resistance, $I_b - V$ characteristics exhibit a finite slope instead of a plateau below the switching current of the junction.

In Setup I2 a bias current I_b is applied between one probe and the drain. A voltage drop V is

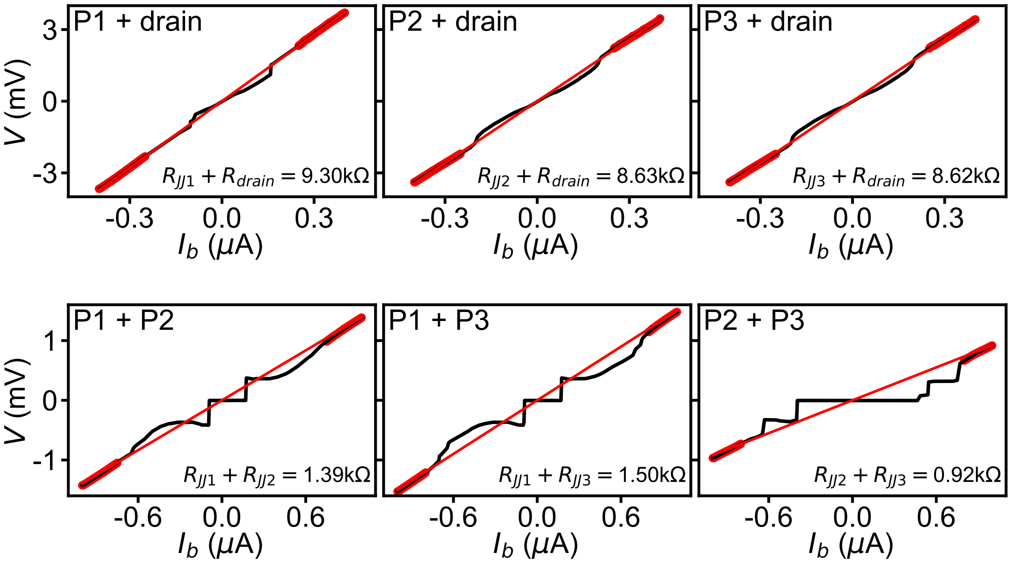

Figure S4: **Contact resistance of the drain in Device 3**: $I_b - V$ characteristics (black) obtained in current-bias measurements of Device 3 at zero field. The indicated pairs of leads are connected as in Setup I1. By fitting the characteristics at high bias (red) with linear functions, series resistances of two Josephson junctions or of one Josephson junction and the drain are obtained and denoted in the bottom-right corners. The resistances of the three Josephson junctions and the resistance of the drain contact are estimated to be: $R_{JJ1} \sim 1.1\,\mathrm{k\Omega}$, $R_{JJ2} \sim 0.4\,\mathrm{k\Omega}$, $R_{JJ3} \sim 0.5\,\mathrm{k\Omega}$ and $R_{drain} \sim 8.2\,\mathrm{k\Omega}$,.

measured between the probe and its first neighboring probe. The remaining probes are floating. Fig. S3e shows Setup I2 with the bias current applied to the probe P1, and the voltage drop measured between P1 and P2. Note that the current in P2 is zero and that the measured voltage drop corresponds to the voltage drop only across the Josephson junction of the probe that is current-biased (JJ1). Upon disconnecting P1 and P2 in Fig. S3d, bias current can analogously be applied in the same setup to any other probe. In contrast to Setup I1, $I_b - V$ characteristics exhibit a zero-voltage plateau below the switching current of the junction - as the voltmeter measures only the voltage drop across the junction.

Device 3 is first characterized at zero magnetic field and zero gate voltages by measuring

$I_b - V$ characteristics in current-bias measurements. First, each superconducting probe (P1, P2 and P3) is connected with the drain contact as in Setup I1 (see the top row in Fig. S4). Then, each pair of the superconducting probes is connected as in Setup I1 (see the bottom row in Fig. S4). The six measured $I_b - V$ characteristics reveal a residual resistance of $R_{drain} \sim 8.2\,\mathrm{k\Omega}$ in the drain contact, as shown in Fig. S4. Therefore, the current-bias measurements of Device 3 are performed in Setup I2, such that voltage drops developed across the drain contact are not measured. The residual resistance R_{drain} can be attributed to an incomplete removal of AlOx by the Ar ion milling during the ex-situ fabrication of the drain contact. In order to avoid voltage divider effects due to the residual drain resistance, the conductance measurements of Device 3 are performed only in Setup V1. Since the residual drain resistance is much smaller than the subgap resistance of the tunnel probes (hundreds of $\mathrm{k\Omega}$), applied bias voltages predominantly drop across the tunnel probes, and tunneling spectroscopy can reliably be performed in Setup V1.

Tunnel gate of Device 3 was not functional as it had a leakage to the nanowire - likely due to the Ar ion milling damaging the $\mathrm{HfO_2}$ gate dielectric. Therefore, the tunnel gate of Device 3 was floating in all measurements.

Extended data

Below we show additional measurement results that reproduce or supplement our findings presented in the main text.

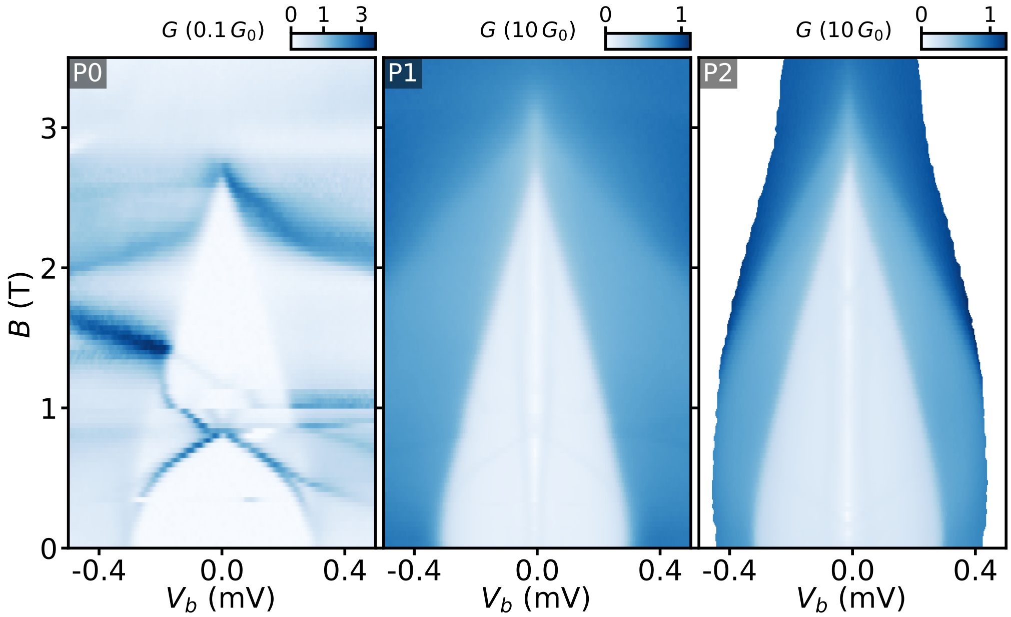

Figure S5: **Characterization of the tunnel probes by differential conductance measurements (Device 2)**: G as a function of V_b and **B** measured by probes P0, P1 and P2. First, probes P0 (left) and P1 (middle) are connected as in Setup V3, and then probes P1 and P2 (right) are connected as in Setup V3. Probe P3 of Device 2 is not functional. The gate voltages are $V_{TG} = 0.75\,\mathrm{V}$ and $V_{SG} = 0\,\mathrm{V}$. Subgap states in P1 and P2 cannot be resolved due to the sensitivity of the lock-in amplifier being adjusted to measure high out-of-gap conductance in these probes, similar as in Fig. 2.

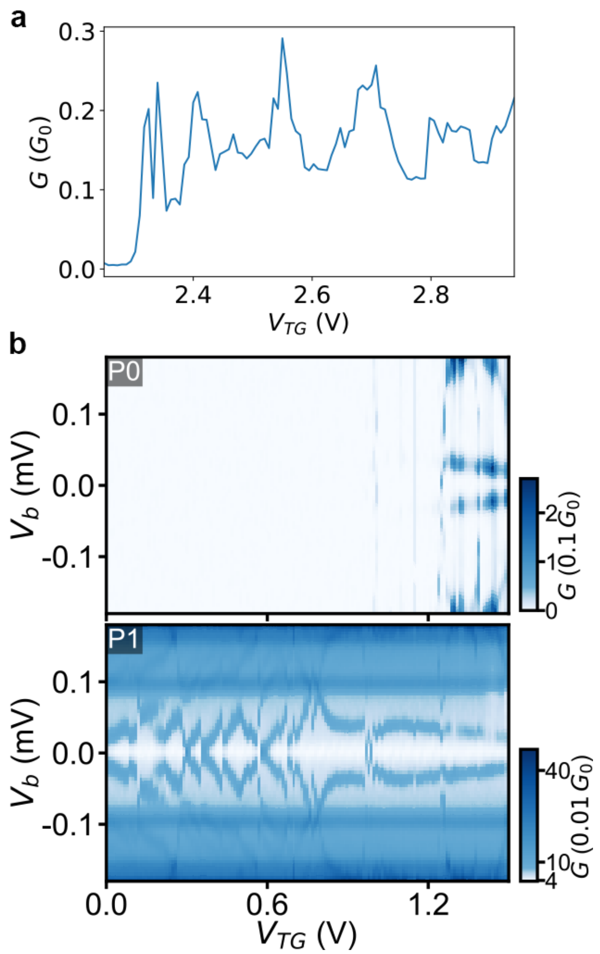

Figure S6: **Effect of the tunnel gate on the tunneling spectroscopy by probes P0 and P1 (Device 1)**. **a** G as a function of V_{TG} for P0 at a bias voltage of $-0.6\,\mathrm{mV}$ with the super gate and other probes floating. The data is measured as in Setup V1. We would note that the pinch-off trace was taken with different parameter settings from panel (b). In spite of this, the panel shows that the tunnel gate is able to tune the semiconducting junction from a relatively open regime to a tunneling regime. **b** G as a function of V_b and V_{TG} measured by probes P0 and P1 connected as in Setup V2. A magnetic field of $0.23\,\mathrm{T}$ is applied perpendicular to the substrate - such that a subgap states is detected by P0 and P1- and $V_{SG} = 0.6\,\mathrm{V}$. For V_{TG} above $\sim 1.2\,\mathrm{V}$, the semiconducting junction of probe P0 is conductive and the subgap state is detectable by both probes. The same state can be detected by P1 while the junction of P0 is pinched-off.

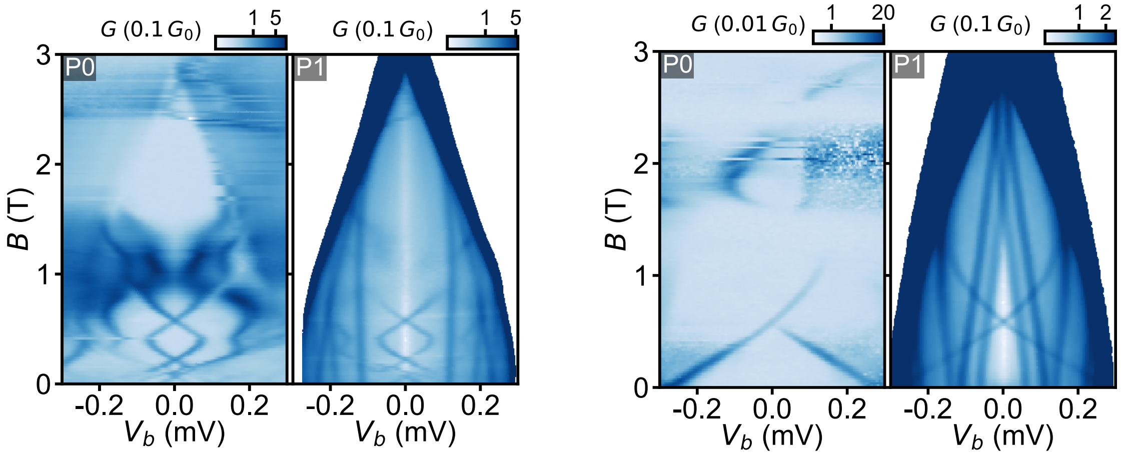

Figure S7: **Comparison between the tunneling spectroscopy by probes P0 and P1 with different parameter settings (Device 1)**: G as a function of V_b and **B** measured by probes P0 and P1 at $V_{SG} = 0.6\,\text{V}$ and $V_{TG} = 1.5\,\text{V}$ (left two panels) and at $V_{SG} = 0.6\,\text{V}$ and $V_{TG} = 2.12\,\text{V}$ (right two panels). The left two panels are measured as in Setup V2 and the right two panels are measured as in Setup V3.

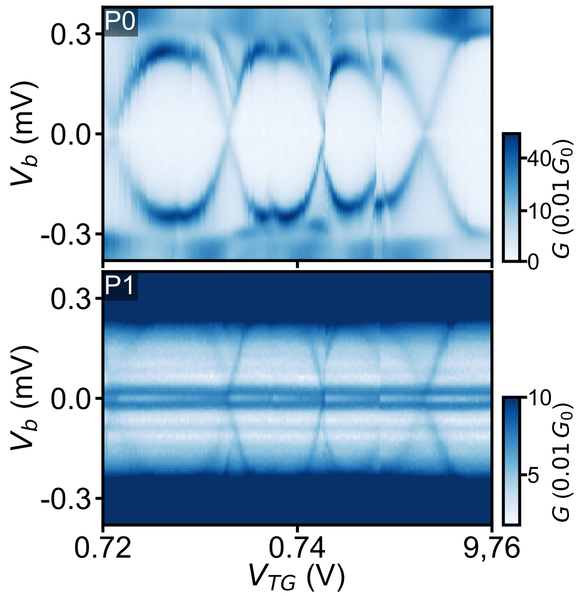

Figure S8: **Comparison between the tunneling spectroscopy by probes P0 and P1 (Device 2)**: G as a function of V_b and V_{TG} measured by probes P0 and P1 connected as in Setup V3, at $\mathbf{B} = 0\,\text{T}$ and $V_{SG} = 0\,\text{V}$. Subgap states sensitive to the tunnel gate are detectable by both probes. Subgap states insensitive to the tunnel gate are only detectable by P1.

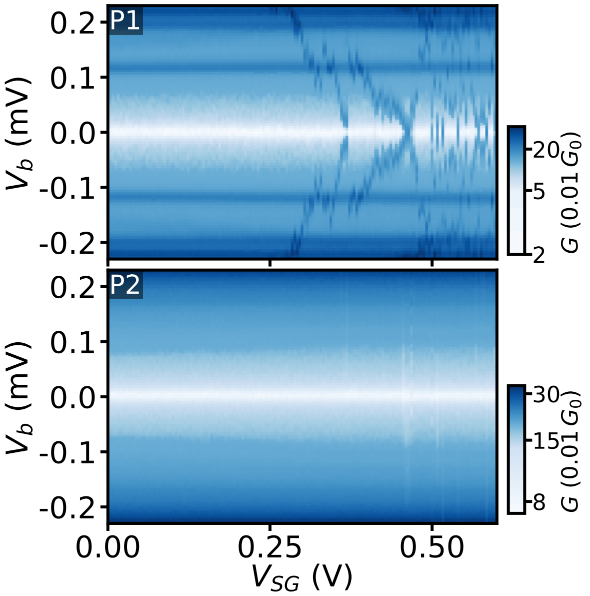

Figure S9: **Effect of the super gate on the tunneling spectroscopy by probes P1 and P2 (Device 1)**: G as a function of V_b and V_{SG} measured by probes P1 and P2 connected as in Setup V2. The tunnel gate is floating and $\mathbf{B} = 0.34\,\mathrm{T}$. For V_{SG} above $\sim 0.4\,\mathrm{V}$, subgap states sensitive to the super gate are detected by P1. Also, subgap states insensitive to the super gate are detected by P1. None of these states are detectable by P2.

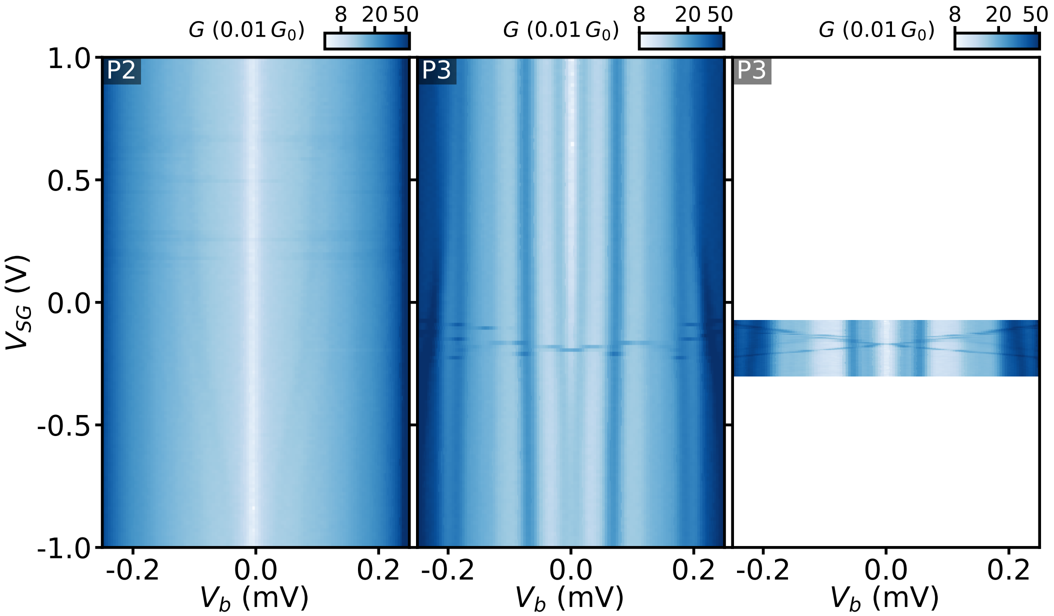

Figure S10: **Effect of the super gate on the tunneling spectroscopy by probes P2 and P3 (Device 1)**: G as a function of V_b and V_{SG} measured by probes P2 (left) and P3 (middle) connected as in Setup V2. The tunnel gate is floating and $\mathbf{B} = 0.5\,\mathrm{T}$. A narrow V_{SG} range is remeasured by P3 in higher resolution (right). No subgap states are detected by P2. Subgap states weakly sensitive to the super gate and a single subgap state highly sensitive to the super gate are detected only by P3.

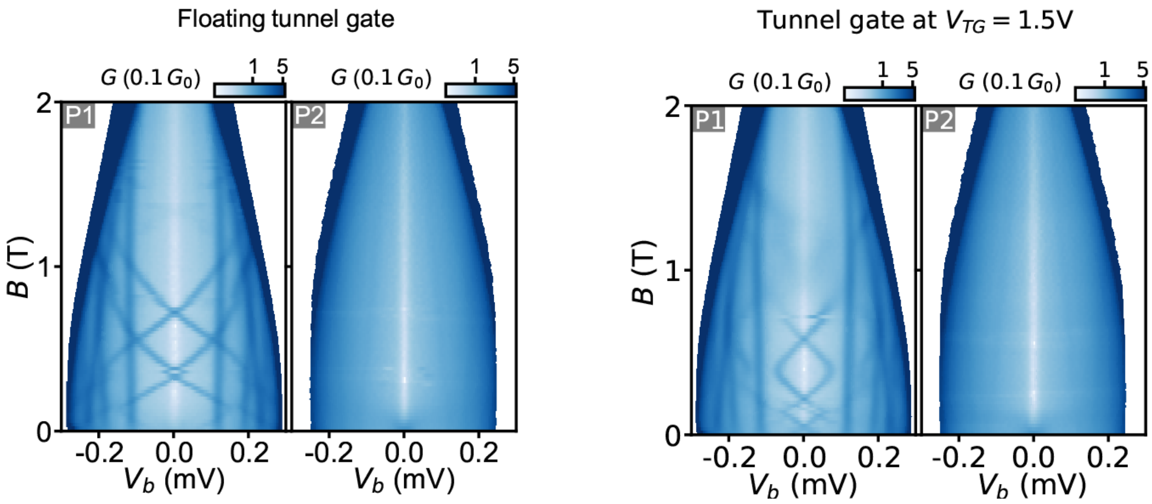

Figure S11: **Effect of the tunnel gate on the tunneling spectroscopy by probes P1 and P2 (Device 1)**: G as a function of V_b and $\mathbf{B}$ measured by probes P1 and P2 connected as in Setup V2, at $V_{SG} = 0.6\,\mathrm{V}$. The tunnel gate is floating (left) or set to $V_{TG} = 1.5\,\mathrm{V}$ (right). Only the subgap states with high g factor at the end of the hybrid are sensitive to the tunnel gate regime. All other properties of the spectra are not affected by the tunnel gate regime.

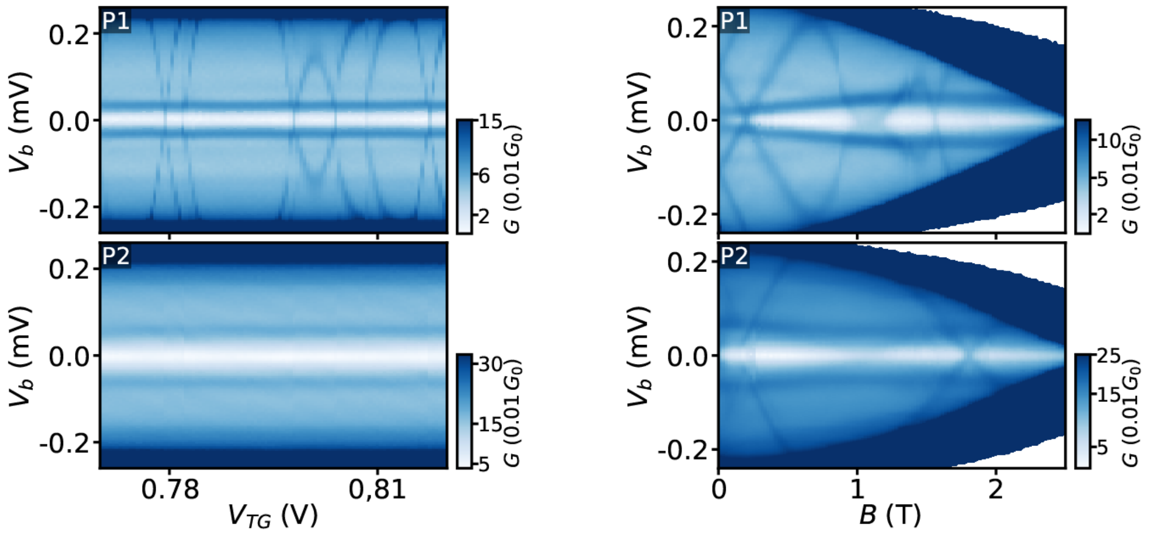

Figure S12: **Effect of the tunnel gate and parallel magnetic field on the tunneling spectroscopy by probes P1 and P2 (Device 2)**: G as a function of V_b and V_{TG} (left) or $\mathbf{B}$ (right) measured by probes P1 and P2 connected as in Setup V3, at $V_{SG} = 0\,\mathrm{V}$. **B** is fixed at $0.6\,\mathrm{T}$ in the gate sweep and V_{TG} is fixed at $0.81\,\mathrm{V}$ in the field sweep. Multiple subgap states with high g factor are detected by single probes. A subgap state tunable by the tunnel gate is detected only by P1. This implies that all the subgap states are localized within $\sim 200\,\mathrm{nm}$ along the hybrid.

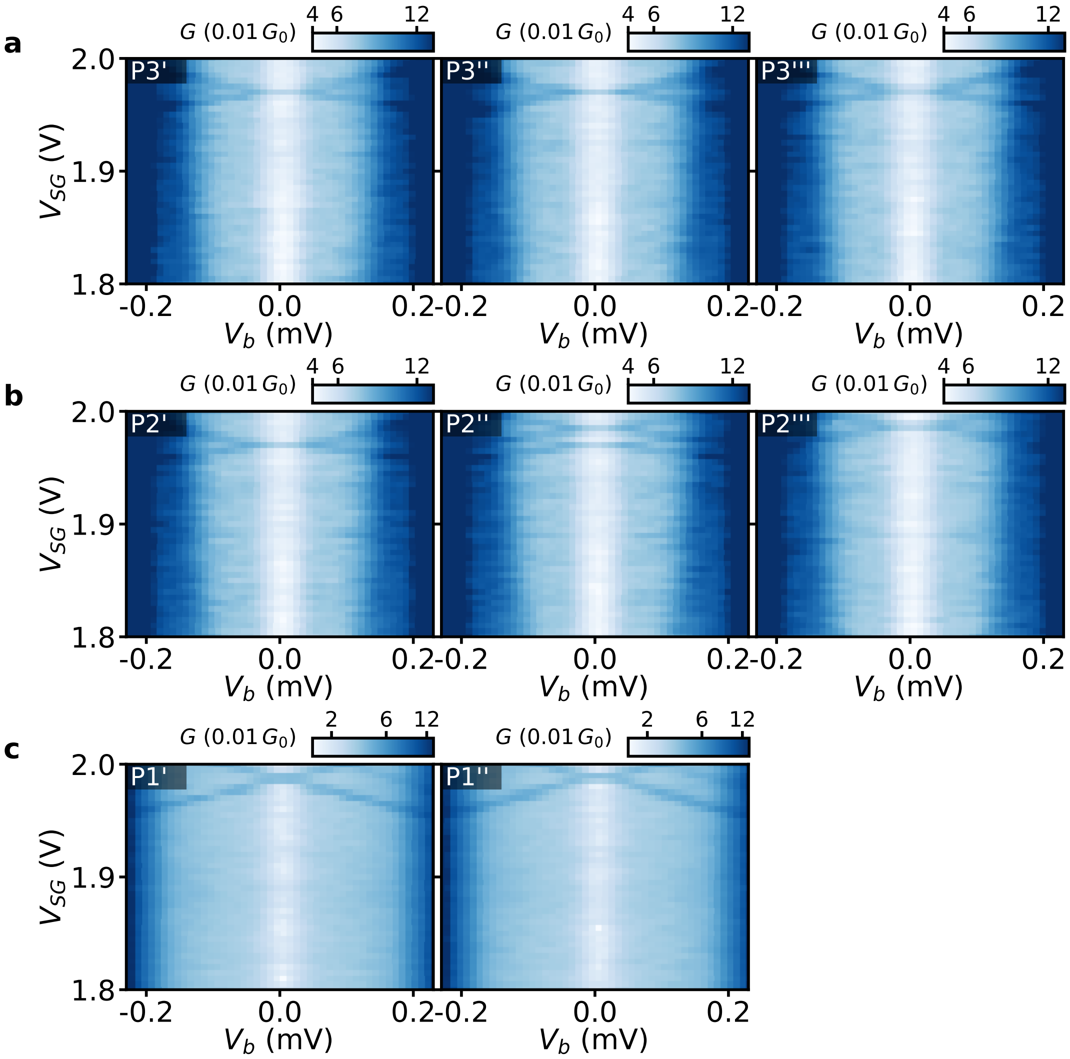

Figure S13: **An example of a subgap state detectable by multiple probes (Device 3)**: G as a function of V_b and V_{SG} measured by probes: (**a**) P3, (**b**) P2 and (**c**) P1 consecutively connected as in Setup V1, at $\mathbf{B} = 1\,\mathrm{T}$. The measurements are performed in the order P3-P2-P1. The super gate is swept over the same voltage range multiple times for each probe in order to check the charge stability of the electrostatic environment (repeated measurements are shown in the same row of (a-c)). A single subgap state is detected by both P3 and P2. This state is sensitive to a charge jump observed in the second measurement by P2. The same state appears shifted in the third measurement by P2. A subgap state in the same V_{SG} range is detected by P1. However, the lever arm of the super gate in this measurement is different – meaning that the subgap state detected by P1 may be different from the one detected by the other two probes.

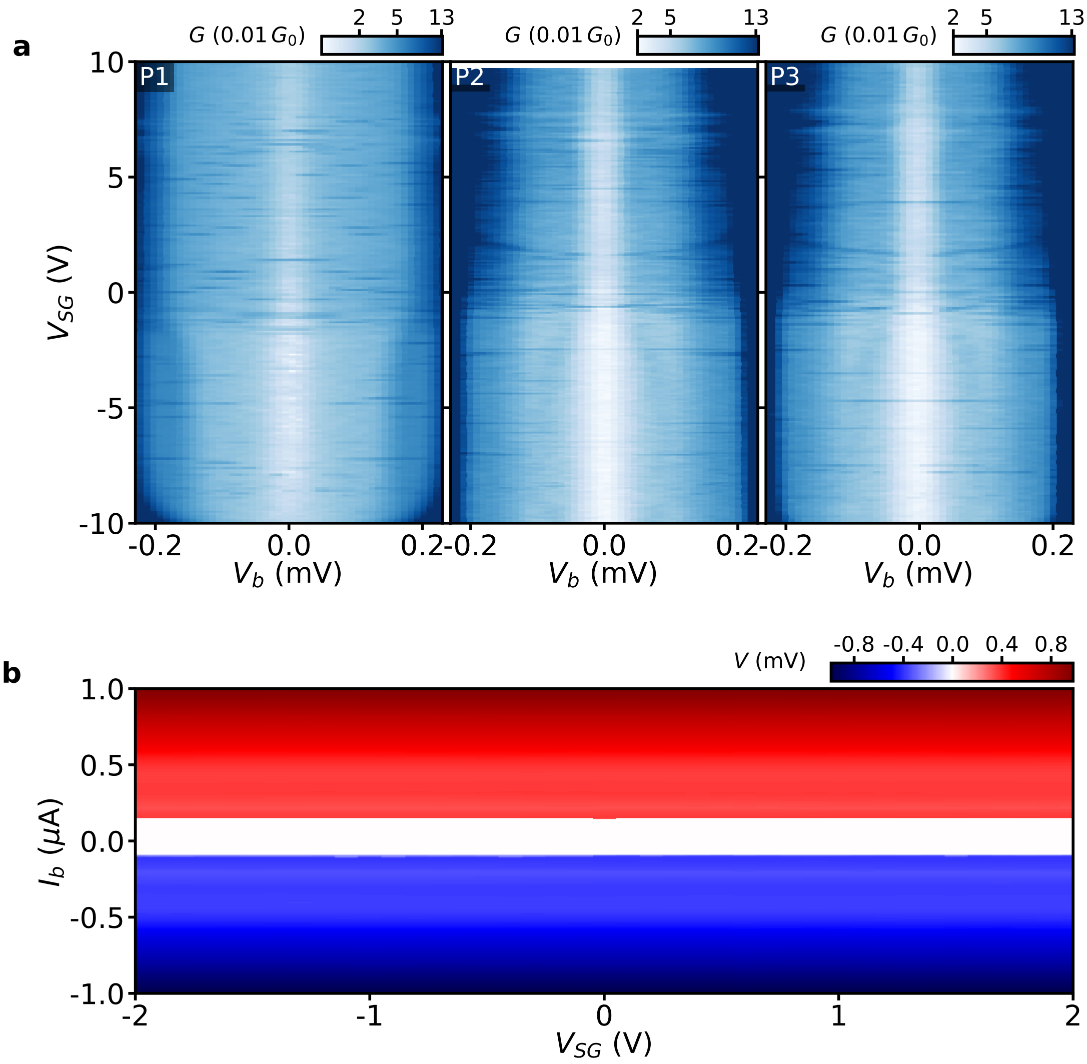

Figure S14: **Effects of the super gate over broad voltage ranges (Device 3)**. **a** G as a function of V_b and V_{SG} measured by probes P1, P2 and P3 consecutively connected as in Setup V1. The tunnel gate is floating and $\mathbf{B} = 1\,\mathrm{T}$. **b** V as a function of I_b and V_{SG} at zero magnetic field. Probes P1 and P2 are connected as in Setup I2, with the bias current applied to P1.
